# Pseudo-HE images derived from CARS/TPEF/SHG multimodal imaging in combination with Raman-spectroscopy as a pathological screening tool

**DOI:** 10.1186/s12885-016-2520-x

**Published:** 2016-07-26

**Authors:** Thomas W. Bocklitz, Firas Subhi Salah, Nadine Vogler, Sandro Heuke, Olga Chernavskaia, Carsten Schmidt, Maximilian J. Waldner, Florian R. Greten, Rolf Bräuer, Michael Schmitt, Andreas Stallmach, Iver Petersen, Jürgen Popp

**Affiliations:** 1 Institute of Physical Chemistry and Abbe Center of Photonics, Friedrich-Schiller University Jena, Helmholtzweg 4, Jena, Germany; 2 Leibniz-Institute of Photonic Technology, Albert-Einstein-Str. 9, Jena, 07745 Germany; 3 Iraqi Centre for Cancer and Medical Genetics Research, Al-Mustansiriya University, Baghdad, Iraq; 4 Institute of Pathology, University Hospital - Friedrich Schiller University Jena, Ziegelmühlenweg 1, Jena, D-07743 Germany; 5 Clinic for Internal Medicine IV, Jena University Hospital, Jena, 07747 Germany; 6 Department of Medicine 1, Friedrich-Alexander-University, Erlangen, 91054 Germany; 7 Erlangen Graduate School in Advanced Optical Technologies (SAOT), Friedrich-Alexander Universität Erlangen-Nürnberg, Erlangen, Germany; 8 Institute for Tumor Biology and Experimental Therapy, Georg-Speyer-Haus, Paul-Ehrlich-Straße 42-44, Frankfurt, 60596 Germany

**Keywords:** Cancer detection, Multimodal imaging, Pseudo HE-images, Raman spectroscopy

## Abstract

**Background:**

Due to the steadily increasing number of cancer patients worldwide the early diagnosis and treatment of cancer is a major field of research. The diagnosis of cancer is mostly performed by an experienced pathologist via the visual inspection of histo-pathological stained tissue sections. To save valuable time, low quality cryosections are frequently analyzed with diagnostic accuracies that are below those of high quality embedded tissue sections. Thus, alternative means have to be found that enable for fast and accurate diagnosis as the basis of following clinical decision making.

**Methods:**

In this contribution we will show that the combination of the three label-free non-linear imaging modalities CARS (coherent anti-Stokes Raman-scattering), TPEF (two-photon excited autofluorescence) and SHG (second harmonic generation) yields information that can be translated into computational hematoxylin and eosin (HE) images by multivariate statistics. Thereby, a computational HE stain is generated resulting in pseudo-HE overview images that allow for identification of suspicious regions. The latter are analyzed further by Raman-spectroscopy retrieving the tissue’s molecular fingerprint.

**Results:**

The results suggest that the combination of non-linear multimodal imaging and Raman-spectroscopy possesses the potential as a precise and fast tool in routine histopathology.

**Conclusions:**

As the key advantage, both optical methods are non-invasive enabling for further pathological investigations of the same tissue section, e.g. a direct comparison with the current pathological gold-standard.

## Background

The WHO expects the annual incidences of cancer to almost double to 21.6 million by 2030 [1]. Evidently, an early cancer diagnosis is the key factor for the survival of patients. The diagnosis of cancer after initial suspicion is complex and involves a number of elaborated diagnostic approaches such as genomics and proteomics [2–4]. A histopathological examination of the excised tissue is the current gold-standard for deriving the final diagnosis [5]. In the majority of cases, the pathologist works with fixated and embedded tissue samples. In order to save time, also native frozen sections are evaluated as part of a quick frozen section analysis. The analysis of frozen sections, however, is challenging and sometimes deviates from the results of an analysis of fixated and embedded sections [5]. To overcome the current limitation of frozen section diagnostics, new pathological tools are required allowing for fast and accurate *ex corpore in vivo* diagnosis of malignant transformed tissue. Thereby, the term *ex corpore in vivo* refers to fresh biopsy tissue. Ideally, these techniques are also applicable for in vivo investigations adding the necessity to work non-invasive, i.e. preserving the tissue’s integrity.

Within the last years the development and application of optical methods for clinical pathology that potentially meet these requirements has rapidly increased [5]. Among these methods, spectroscopic imaging approaches are of particular significance [6–9]. *Ex vivo* reflectance confocal microscopy (CRM) was used to detect residuals of non-melanoma skin cancer (NMSC) during Moh’s surgery [10]. Optical coherence tomography (OCT) demonstrated its potential to differentiate between malignant and benign tissue areas in head and neck, skin, genital and bladder cancer [11]. The potential of photo acoustic imaging (PAI) for cancer diagnostics was evaluated for melanoma [12] and breast cancer [13]. One-photon excited autofluorescence (OPEF) was utilized to investigate fibrosarcoma, lung cancer and NMSC [14]. Linear Raman-microscopy was applied for differentiation of healthy tissue from cancerous epithelium within human skin, colon, brain and breast [15]. Further, various non-linear microscopy methods were shown to enable for detection of NMSC as well as head and neck, brain or lung cancer [16]. However, most of these studies represent proof-of-concept studies and except of OCT these methods have not been transferred into routine clinical applications. The delay of technology transfer may be attributed – among other reasons – to the increasing complexity of state of the art cancer diagnostics reducing physician’s available time to familiarize with new imaging technologies as well as its image contrast and significance. Thus, it is the task of scientists to reduce the complexity of new technologies by translating the image information into a format that physicians are accustomed to such as hematoxylin and eosin (HE) stained images or by direct prediction of the tissue’s malignancy state. Ideally, this image translation is achieved entirely by computational image analysis requiring no assistance of scientists or physicians.

To automate such a translation of optical microscopy data, the image contrast is required to provide cancer specific information in the first place. Since the information transferred by a single modality is limited, various optical methods are frequently combined such as Raman-spectroscopy and OCT for skin cancer detection [17]. These multimodal approaches can be grouped into those that gather techniques with similar image acquisition times and experimental equipment and combinations that merge sensitivity with specificity by coupling fast imaging tools with techniques of high information density per pixel. Here, we combine both multimodal concepts to maximize the image acquisition speed and information depth in order to improve the accuracy of image translation and diagnostic results.

First, the fast non-linear microscopy methods CARS = coherent anti-Stokes Raman-scattering, SHG = second harmonic generation and TPEF = two-photon excited autofluorescence were jointly applied to characterize the architecture and biochemical composition of frozen tissue sections. For the selection of regions of interest (ROI), we demonstrate for the first time the possibility to derive computationally pseudo-HE images from CARS/SHG/TPEF-images by applying multivariate statistics. The pseudo-HE image can be analyzed by a pathologist in the same manner as a normal HE image. Following the selection of ROI based on pseudo-HE images we applied Raman-spectroscopy for the prediction of the diagnosis. Though compromised by its poor sensitivity, Raman-spectroscopy is unprecedented for its high specificity yielding information based on inherent molecular vibrations that - like fingerprints - specifically characterize chemical structures and biochemical compositions of e.g. biological cells, tissues etc.

The non-invasiveness of non-linear multimodal imaging and Raman-spectroscopy enables for the direct comparison with the pathological gold standard requiring staining. Thus, further analyses can be performed on the samples pre-characterized by combining multimodal imaging and Raman-spectroscopy. This new approach, the combination of non-linear multimodal images and Raman-spectra of selected regions, was evaluated for *ex vivo* sections of colon tissue that arose from p53 knockout mice [18]. The mouse model was selected to investigate a single type of tumor while minimizing the variance between individuals (mice). The results, therefore, allow for a reliable estimation of the generalizability of the proposed optical pathological tool with respect to the adenoma-carcinoma-sequence and its diagnostic value.

## Methods

### Sample preparation

Mice with an intestinal epithelial cell (IEC)-specific deletion of the tumor suppressor gene p53 were received by crossing of floxed p53 mice with villin-Cre mice (Tp53 ^*Δ*IEC^) [19]. As controls Tp53 ^F/F^ mice were utilized. These mice were treated intraperitoneally once a week for 6 weeks with the carcinogen azoxymethane (AOM; 10 mg/kg). For histological and Raman-spectroscopic investigations mice were sacrificed at different time points after the AOM application. The specific loss of p53 in the intestine markedly enhances the carcinogen-induced tumor incidence and leads to the development of invasive colorectal tumors beginning about 12 weeks after the first AOM treatment.

Full preparation of colon and rectum was performed for 47 individuals. Biopsies were acquired from 8 out of these 47 individuals. Another cohort of 22 individuals was divided into two groups of which biopsies were taken in an alternating fashion every two weeks. In total, 69 individuals contributed to the complete training Raman-dataset. For the present study, 6 individuals (2 males, 4 females) were selected randomly covering the whole spectrum of diagnoses. In total 34 Raman-maps (46240 spectra) and the respective non-linear multimodal images were acquired to investigate this reduced cohort.

Cryosections for the histo-pathological evaluation were obtained following the standard procedure of washing the specimens with phosphate buffered saline (PBS Buffer), fixation using paraformaldehyde, embedding in paraffin and cutting the sample in 10 *μ*m slices which were stained with hematoxylin and eosin (HE staining). For the Raman-spectroscopic analysis, the specimens were washed with PBS buffer and immediately frozen in liquid nitrogen. Distilled water was used as embedding medium for the cryosectioning step resulting in 20 *μ*m thick slices. Thus, a native sample - preserving the lipid distribution - is obtained.

Brightfield images of the parallel HE stained as well as Raman-spectroscopically examined sections were recorded using a halogen illuminated Leica DM2500 microscope (Leica Microsystems, Wetzlar, Germany) equipped with a Nikon Digital Sight (DS) camera system using the DS-Fi1 CCD camera head (Nikon Instruments Europe B.V., Germany). To superimpose the measured Raman-maps with the their corresponding histopathological assessment, all HE stained scan areas were imaged using five different Leica objectives in the range of 1.25 × to 40 × (1.25 × HCX PL Fluotar (NA 0.04), 5 × N Plan (NA 0.12), 10 × N Plan (NA 0.25), 20 × N Plan (NA 0.4), 40 × N Plan (NA 0.65)).

### Non-linear multimodal microscopy

**Fig. 1:**
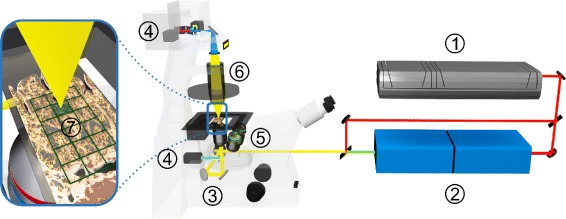
Schematic of the experimental setup used for non-linear multimodal microscopy. *1* Ti:Sa-laser; *2* Optic parametric oscillator (OPO); *3* Rotating mirror; *4* Photomultiplier (PMT); *5* Objective lens; *6* Condenser; *7* sample with superimposed grid outlining individual squares of the composite multimodal image

The experimental setup was presented elsewhere [20]. A schematic of the experimental setup is displayed in Fig. 1. Briefly, a Coherent Mira HP Titanium-Sapphire (Ti:Sa) laser (Coherent, Santa Clara, USA) is pumped by a continuous wave Neodymium-Vanadate laser with an average power of 18 W operating at 532 nm. The Ti:Sa-laser generates 2–3 ps pulses (FWHM) at 830 nm with a repetition rate of 76 MHz. The 3.5 W averaged output power of the Ti:Sa-laser is split into two parts. The first part is used directly, i.e., without frequency conversion, as the Stokes beam. The second part is coupled into an optical parametric oscillator (OPO, APE, Berlin, Germany) that allows to adjust the pump wavelength in the range from 500 to 1600 nm. To match the CH _2_ symmetrical stretching vibration at 2850 cm ^−1^ for the CARS measurements, the OPO is tuned to 671 nm. Both beams, pump and Stokes, are temporally and spatially overlapped and coupled into a laser scanning microscope (LSM 510 Meta, Zeiss, Jena, Germany) and focused onto the sample with a 20 × (NA 0.8) achromatic objective (Zeiss). The optical non-linear response of the sample is wavelength filtered by means of various dielectric filters and detected by photomultiplier tubes (PMT, Hamamatsu Photonics, Hamamatsu, Japan) in forward (CARS, SHG) and backward direction (TPEF). Large area scans of the samples of up to 15 ×15 tile-scans, each having a size of 450 *μ*m × 450 *μ*m, were recorded. Every tile-scan was acquired with a resolution of 1,024 x 1,024 pixels and a pixel time of 1.6 *μ*s. By averaging twice the total acquisition the time per tile-scan does not exceed 16 s for all three modalities, i.e. CARS, TPEF and SHG. Thus, the acquisition time for an image of 15 × 15 squares corresponding to a size of 6.75 mm × 6.75 mm is about 1 hour. The average power at the sample was 25 mW and 50 mW for the pump and Stokes beam, respectively. A discussion about the applied power and potential linear as well as non-linear tissue photo-damage can be found elsewhere [21].

### Linear Raman-microspectroscopy

Raman-spectra of 20 *μ*m thick cryosections from colon and rectum of mice as well as from biopsies on CaF _2_ slides were recorded with an upright micro-Raman-setup (CRM 300, WITec GmbH, Ulm, Germany) equipped with a 300 g/mm grating (7 cm ^−1^ resolution) and a Deep Depletion CCD camera (DU401 BR-DD, ANDOR, 1024 × 127 pixels) cooled down to −65°C. The tissue Raman-spectra were recorded with a 785 nm diode laser which was focused through a Zeiss 50 × objective (NA 0.7) onto the sections. For the herein described study 34 Raman-scans were recorded in scanning mode with a step size of 5 *μ*m and an integration time of 2 s per spectrum. The scan dimension was chosen to be around 34×40 pixels (170 *μ*m × 200 *μ*m), so every scan consists of approximately 1360 Raman-spectra. As a standard for the subsequent processing of the spectra a time series comprising 50 spectra of 4-acetamidophenol was collected with an integration time of 1 s per spectrum. Based on HE stained parallel sections a trained pathologist selected the areas to be measured. Here we measured the Raman-spectra before the multimodal images in order to check if burning effects occurred. In the presented data set burning effects were not observed.

### Ethical approval

All animal studies were approved by the governmental commission for animal protection (No. 02-007/13).

## Results

### Generation of pseudo-HE images out of CARS/TPEF/SHG multimodal images

**Fig. 2:**
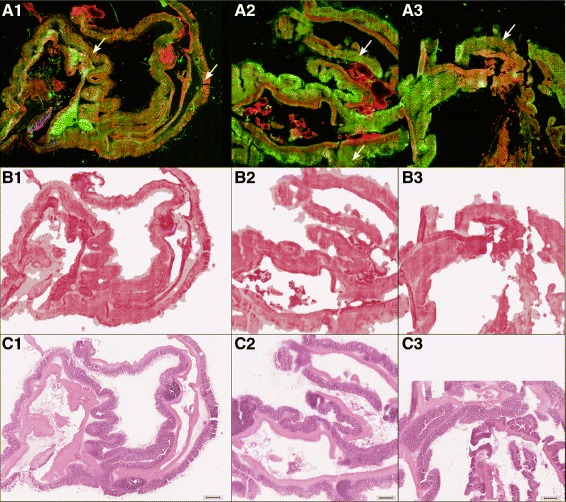
Overview of acquired and generated images of mouse colon sections (3 out of 22 images in total): In *row A*, multimodal images are displayed (for details see Methods Section, non-linear multimodal microscopy). In *row B* and *C* the computationally derived pseudo-HE stained images based on the multimodal images and the HE stained image are displayed, respectively. The pseudo-HE images of *row B* are generated non-invasively allowing for a subsequent analysis by other modalities or stains. Red flag regions, which were subsequently analyzed by Raman-spectroscopy (see Fig. 4) are marked with a white arrow in *row A*. The scale bar represents 500 *μ*m

**Fig. 3:**
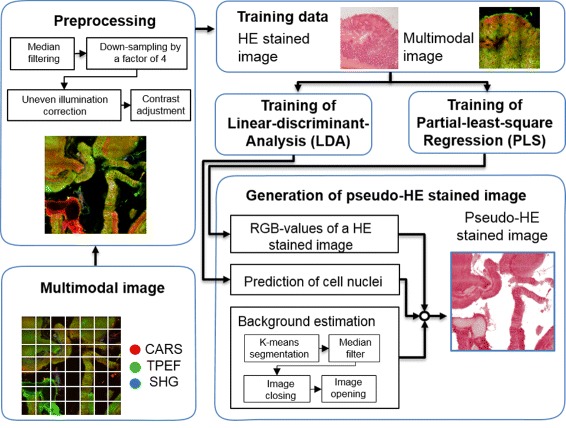
Schematic of the pseudo-HE image generation algorithm. CARS, TPEF and SHG images of the samples were acquired and combined into multimodal images using a RGB color model. The multimodal images were preprocessed as follows. First, noise was removed by median filtering and the images were down-sampled by a factor of 4. After that the uneven illumination of the single tile-scans was corrected and the contrast was adjusted. For generation of the pseudo-HE stained images, a RGB color image and two masks were calculated. To convert a multimodal image into RGB values of pseudo-HE image, a partial-least-square regression (PLS) method with 3 components was used which was trained with one image. The mask of cell nuclei was predicted by a pre-trained LDA model and was used to color cell nuclei regions within the pseudo-HE stained image in dark violet. Moreover, a background mask was estimated and used to color the background area of the pseudo-HE image in white

22 multimodal images were measured and the TPEF, CARS and SHG channel were combined in false-colors. Here we used green to represent TPEF, red for CARS and SHG was coded in shades of blue. The images were pre-treated separately: First the images were down-sampled by a factor of 4. Then the uneven illumination of the tile-scans was removed within the images and the contrast was adjusted (CARS:[0.05-0.015], TPEF:[0.05-0.04], SHG:[0.001-0.001]) [22]. The resulting images are given in Fig. 2 in row A. The workflow for pseudo-HE generation is sketched in Fig. 3. The pseudo-HE stained images are generated using a partial least squares regression (PLS) model with 3 components [23], which was trained with one image (data not shown). PLS is a multivariate regression method that estimates the relationship between two datasets and differs from traditional least squares regression in utilizing information of the independent and the dependent variables. The RGB-values of an HE stained image was modeled using the three color channels of the multimodal image. Afterwards, regions with a certain fingerprint of CARS, TPEF and SHG intensities were predicted to feature cell nuclei (dark violet) and thus this color was added. This procedure was based on a linear discriminant analysis model (LDA). The main idea of this classification method is to find the optimal linear combination of variables that maximizes the variations between different classes and minimizes the variations within these classes. This additional model was necessary as cell nuclei had negative contrast in the multimodal images. The result is referred to as computational HE stained or pseudo-HE image. Its generation is performed automatically and, therefore, allows for a fast screening. After the calculation of the computational HE stain was performed, the background was determined and set to white. The background was estimated by a sequence of steps. First the original multimodal image (RGB) was segmented by k-means clustering with k=6. The pixels of the darkest class, i.e. the class with the lowest value of sum over squared class center, are considered as background contribution. Based on this annotation a background mask was calculated. To remove noise contributions in the segmentation result, the estimated background mask was filtered by a median filter. Thereafter morphological closing was applied to fill gaps in the foreground and extending the foreground area. Finally, the background mask was morphologically opened in order to remove small foreground areas and smooth the background edges (or specimen contour). The resulting mask was mean filtered and used as a weighting mask allowing for a smooth removal of large background areas.

### Spectral histopathology — statistical analysis of Raman-spectra

The data preprocessing in case of the Raman-spectra and statistical modeling were performed using the software package R [24]. The packages used were ‘MASS’ [25], ‘pls’ [23], ‘KKNN’ [26] and ‘Peaks’ [27]. Several multivariate statistical tools were applied, such as principal component analysis (PCA), k-means and Weighted k-Nearest Neighbors (KKNN), which shall be described briefly. For dimension reduction and data compression PCA is the most popular and useful tool. The PCA transforms the variables of a dataset into a new set of variables that are linear combinations of the former variables. The new values are called principal components and are ranked according to the variance contributions so that the first principal component provides the highest data variance. Choosing only the first few principal components allows a dimension reduction with a marginal loss of information. K-means is an unsupervised clustering method that arranges the unlabeled dataset into a given number of groups (clusters). It starts with a random distribution of cluster centers and iteratively moves them in order to minimize the total within-cluster variance [28]. KKNN is a non-parametric supervised classification method that is often used because of its simplicity and good performance. To assign a new observation, first the k observations in the training dataset have to be found, which are closest to the new observation. Then the new observation is classified through the majority vote among the k neighbors. KKNN - as an extension of k-Nearest Neighbors algorithm - also takes into account the individual distances of the nearest neighbors to the new observation in the form of weights [26]. The performance of the classification was verified through individual-out-cross-validation (IO-CV).

A standard spectral pre-treatment was applied [29]. First a background was subtracted using the SNIP algorithm [30] followed by a vector normalization. The spectra were projected on a PCA, minimizing the necessary dimensions and computational time. The annotation of a pathologist was transferred to a computer model by a k-means-cluster-analysis with *k* ranging from 9–15 based on the number of scans of one individual (mouse). The annotation was performed in a blinded manner meaning that the pathologist was not biased by the k-means-cluster-analysis plots and utilized only the HE stained images for diagnostics. Following the annotation, every Raman-spectrum was linked to the pathologist’s diagnosis as the major reason for measuring scans and not single Raman-spectra. This procedure is called spectral histopathology (SHP) and described elsewhere in full detail [31, 32]. The Weighted k-Nearest Neighbors (KKNN) model was trained with one neighbor to be used for prediction and the Minkowski distance in combination with the kernel ‘optimal’. The evaluation was done by an individual-out-cross-validation. This approach incorporates the biological variance in the performance estimation. Only with such an approach a robust estimate of how the model will perform in future applications, e.g. the generalization performance of the model, can be derived. Shortly, the Raman-spectra of one individual were excluded from the model building and a classification model for the groups ‘adenoma’, ‘carcinoma’ and ‘normal’ was constructed. The Raman-spectra of the individual currently excluded were predicted using the classifier and the outcome was stored. Accordingly, the Raman-spectra of all individuals were once used as a test set. The result is put together in Table 1. It should be noted that hyperplasia occured only once and the corresponding region was excluded from the dataset.

**Table 1:** Individual-Out-Cross-Validation (IO-CV) of a KKNN model; the model has a mean sensitivity of 100 % for the classification between tumor and normal regions, but the mean sensitivity drops to 80.16 % for a differential diagnosis, e.g. for the classification task normal-adenoma-carcinoma

	Annotated classes			Characteristics	
Predicted class	Adenoma	Carcinoma	Normal	Sensitivity / %	Specificity / %
Adenoma	4	1	0	80	87.5
Carcinoma	3	5	0	62.5	95.2
Normal	0	0	16	100	100

## Discussion

In this contribution we combined two optical imaging approaches for a fast and precise pathological tissue assessment. The first approach is used to generate an unspecific overview. Therefore multimodal imaging quickly generates large tissue images, which were translated into a pseudo-HE image for identification of suspicious areas. These red flag areas were further analyzed in more detail using Raman-spectroscopy to receive a bio-spectroscopic fingerprint of these suspicious areas. With the help of these fingerprints we were able to construct a model for cancer diagnosis achieving a high sensitivity.

### Multimodal imaging – an overview

The first step of the proposed diagnostic workflow (Fig. 3) consisted of recording a multimodal image, which is utilized to derive a pseudo-HE image by multivariate statistics. For exemplification, the first row (A) in Fig. 2 displays multimodal images of mouse colon sections (for sample details see “Methods” Section, sample preparation). False-colors code CARS in red, TPEF in green and SHG in blue. CARS was adjusted to map the CH _2_-distribution outlining mostly lipids, while the CARS signal originating from proteins is weaker as their molecular mass-to-methylene group ratio is comparably decreased. The TPEF signal was collected in the spectral range between 426–490 nm, thereby, highlighting the distribution of strong auto-fluorophores such as elastin, NAD(P)H and keratin, while SHG collected at 415 nm visualizes the fibrous collagen network.

Proving the similarity of the information content of non-linear multimodal and classical HE stained images, we applied multivariate statistics in order to generate a computational HE stain image out of the multimodal dataset. The results are exemplified in row B of Fig. 2. For the prediction of pseudo-HE images, we utilized a model combining partial-least-square (PLS) regression with a linear-discriminant-analysis (LDA) that was trained using a single HE image. Subsequent to all multimodal measurements, the samples were HE stained and imaged. The corresponding HE images are depicted in row C of Fig. 2. Apparently, morphological information are readily retrieved from both set of images, i.e. in the pseudo-HE images derived from the multimodal images (Fig. 2b) and the real HE images (Fig. 2c). Some information, however, is missing in the pseudo-HE images (Fig. 2b). In particular the cell nuclei are at certain areas not optimally resolved possibly due to signal intensity variations resulting in a non-uniform brightness of the images. It shall be noted that nuclei may be resolved in future pseudo-HE images if stimulated Raman scattering at DNA specific Raman resonances is applied featuring the required high signal-to-noise ratio [33]. From Fig. 2b and c differences in the color composition and brightness are visible. For example, the *submucosa* appears darker within the pseudo-HE images than in the corresponding HE images. Further deviations result from the ethanol washing step during the staining procedure removing soluble components. Consequently, some soluble components were imaged by non-linear multimodal microscopy but were removed in advance to the acquisition of the HE images. The advantage of the generated pseudo-HE images is that the measurement is non-invasive, therefore allowing for further analysis. Here, a HE stain was applied afterwards for comparison, but the employment of other stains or measurement modalities is also possible. Due to its speed and non-invasiveness, the pseudo-HE stain can be applied in a cryosection analysis setting or potentially even *in-vivo*. Based on the pseudo-HE images a pathologist can identify or define suspicious areas (red flags). These small red flag areas can be further investigated by various approaches like e.g. immunostains, or by other label-free spectroscopic techniques featuring a higher molecular sensitivity than non-linear multimodal imaging. In this contribution we applied Raman-spectroscopy as a second diagnostic technique, offering molecular fingerprint information. The results of the Raman-study are summarized in the following section.

### Raman-microspectroscopic imaging – diagnosis

**Fig. 4:**
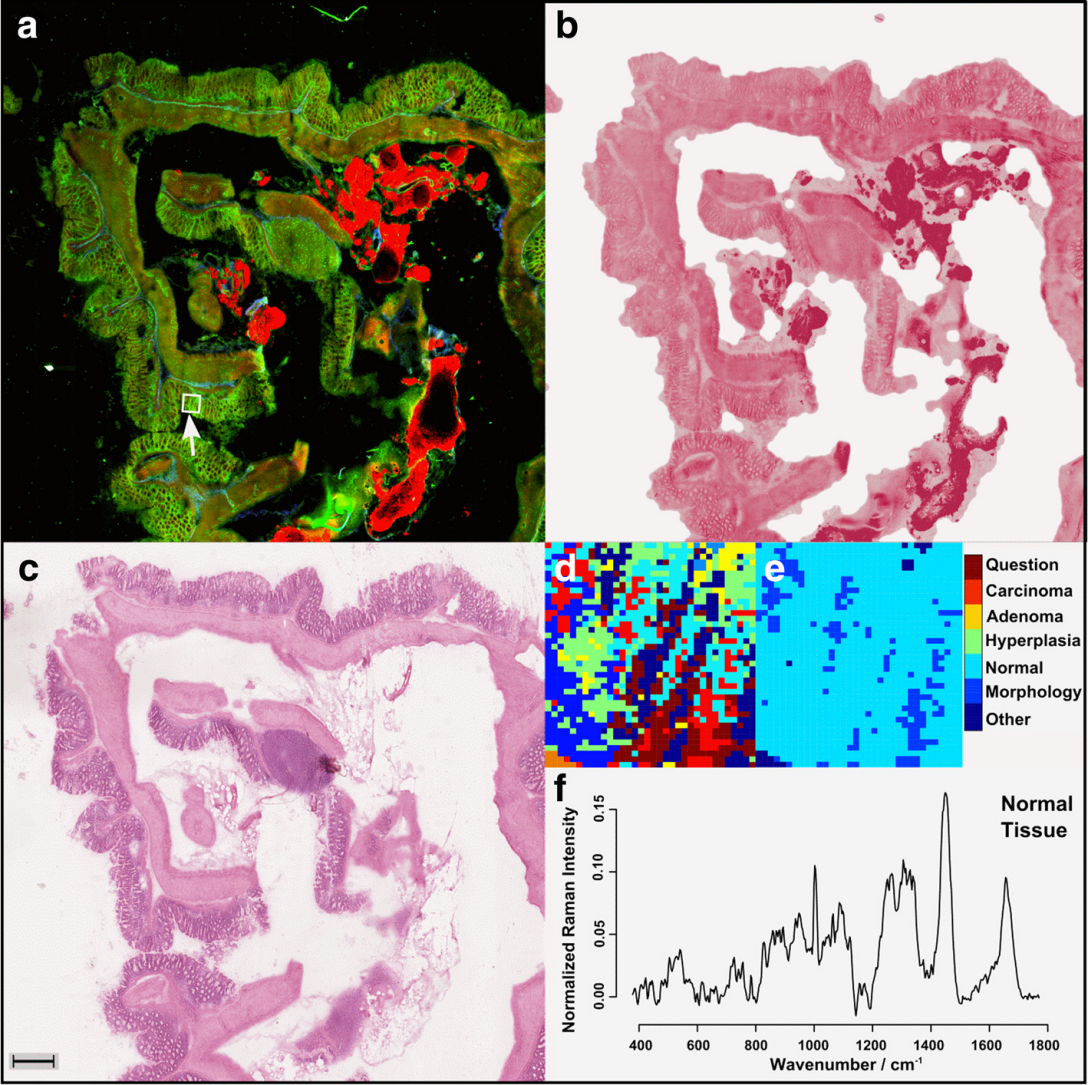
Workflow: **a** Multimodal image of a mouse colon section (for color code see Fig. 2); **b** Pseudo-HE stained image derived from the multimodal image shown in **a**, **c** HE stained image of the same section as investigated in **a**, **d** a k-means-cluster-analysis (*k*=9) of the Raman-measured region, marked with a white arrow in **a**, **e** pathologist’s annotation; Here, normal epithelial tissue with non-epithelial components (other morphological tissue types) and background contributions (*left corner*) are present; **f** pre-treated mean Raman-spectra of the region annotated as normal epithelial tissue in **e**; the scale bar represents 500 *μ*m. The colors within panel **d** were assigned arbitrary due to the k-means clustering, while the legend applies only to panel **e**. The color selection in panel **e** is related to bio-medical information

As mentioned in the previous section certain areas (red flags) were further characterized by means of Raman-spectroscopy. These red flag regions are marked with a white arrow in Figs. 2a and 4a. To prove whether Raman-spectroscopy can be utilized as a molecular selective diagnostic platform, the Raman-spectroscopically characterized regions were annotated and diagnosed by an experienced pathologist in a blinded manner. The adjective ’blinded’ means here that the multimodal images and the Raman-spectroscopic generated image were unknown to the pathologist. The test of the diagnostic value of Raman-spectroscopy was achieved by application of a recently reported workflow termed as spectral-histo-pathology (SHP) [31, 32, 34]. In that approach every Raman-scan is clustered and the diagnosis of the pathologist is transferred to a computer model. Figure 4d displays such a cluster-analysis. This cluster scan is subsequently annotated using the groups displayed in Fig. 4e. Based on this annotation mean Raman-spectra of different regions can be derived. Figure 4f shows as an example the mean Raman-spectrum of normal epithelial tissue. Thereafter, a supervised classification can be applied in order to discriminate between normal, adenoma and carcinoma tissue. A hyperplastic area was only present once in the data set, therefore the corresponding region was excluded from further analysis. In the same manner Raman-spectra of other morphologic areas (muscle, connective tissue) or artefacts (background, spikes) were excluded.

**Fig. 5:**
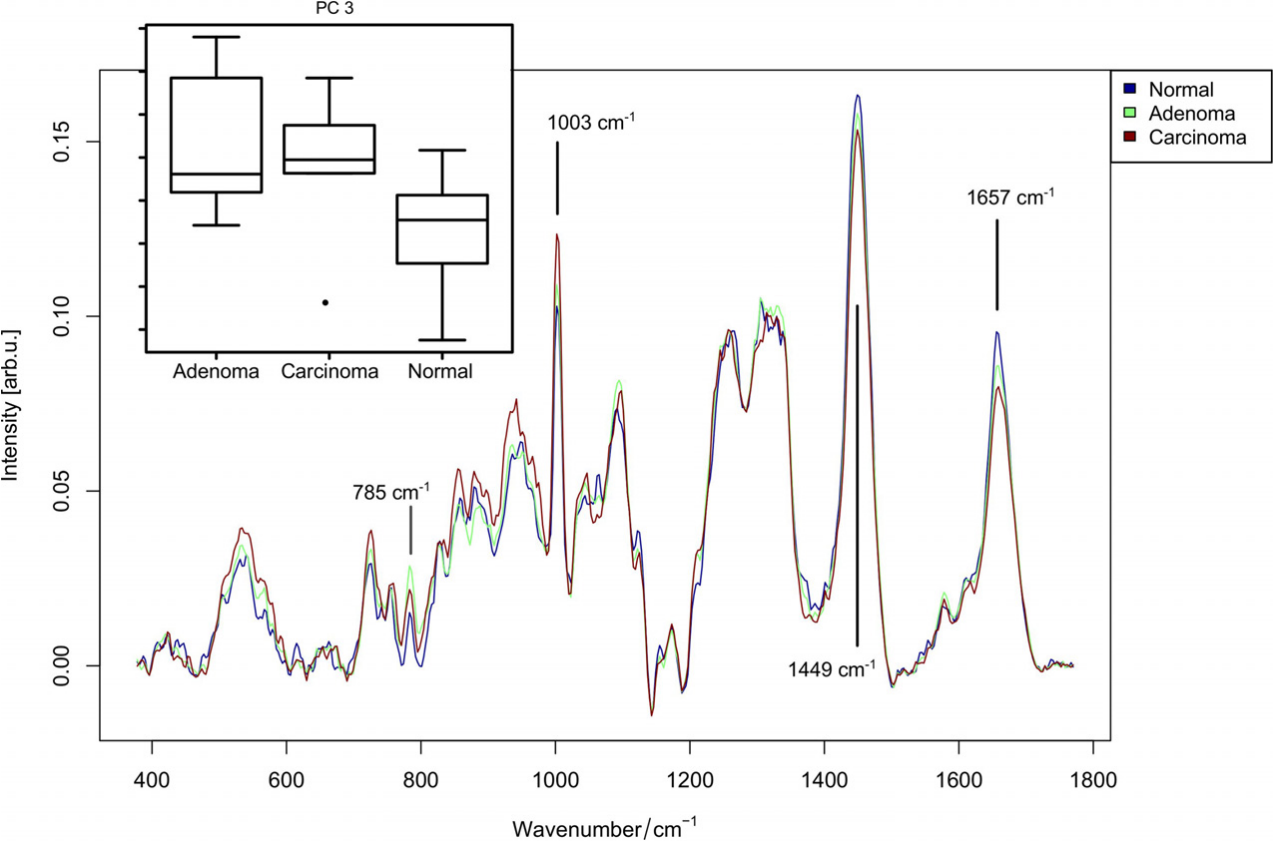
The mean Raman-spectra of adenoma, carcinoma and normal tissue: The mean Raman-spectra corresponding to the classification system are visualized. Selected peaks featuring large difference for distinct classes are marked by gray lines - see text for band assignment. In the upper left corner a boxplot of PC 3 is given, which shows the highest significance (*p* =0.001005) for the separation of normal and tumor tissue

First, a univariate statistical test was applied to verify whether the groups feature statistical significant differences. The statistical significance of the Raman-spectra for the differentiation of tumor against normal tissue and carcinoma against normal tissue were investigated by applying a two-sample Wilcoxon test. The principal component (PC) scores of the components 3, 4 and 5 were proven to be significant for the task normal against tumor tissue. The *p*-values were 0.001005, 0.019 and 0.003, respectively. The scores of the fourth and fifth component were significant for the task ’normal against carcinoma’ (*p*-value 0.027 and 0.00039). A boxplot of the scores of PC 3 is visualized in Fig. 5 together with the mean Raman-spectra of the groups. The band assignment for interpreting these Raman-spectra can be found in a number of publications [35, 36]. Here, we assigned the bands for visualization of the biggest differences and marked them in Fig. 5. The band at 785 cm ^−1^ can be attributed to the phosphate backbone vibration of DNA [35]. The other three bands at 1003, 1449, 1657 cm ^−1^ can be assigned to protein vibrations [35, 36]. The band at 1003 cm ^−1^ originates from the symmetric ring breathing of phenylalanine [35], while the Raman-resonances at 1449 and 1657 cm ^−1^ can be attributed to the CH _2_ deformation vibration [36] and Amide I vibration [35, 36], respectively.

The mean Raman-spectra were subsequently tested for their diagnostic value. Therefore, the Raman-spectra were pre-treated and a Weighted k-Nearest Neighbors (KKNN) classifier was trained and evaluated. In order to estimate the generalization performance of the classifier, the biological variations between the different mice has to be accounted for. To do so, we used an individual-out-cross-validation scheme (IO-CV), where all Raman-spectra of one individual were excluded from training and then predicted. This procedure was iterated and the result is shown in Table. 1. The confusion table shows that the diagnosis of tumor regions, e.g. the classification between tumor regions and normal regions, is accomplished with 100 % mean sensitivity. The differential diagnosis, e.g. the discrimination between adenoma and carcinoma regions, is also possible, but with a lower sensitivity. Here, mis-classifications between adenoma and carcinoma regions occurred. Nevertheless, the overall mean sensitivity for the differential diagnosis is 80.16 % and may be increased in future experiments by improving the detection scheme using, e.g. shifted-excitation Raman-difference-spectroscopy (SERDS) [37]. In a nutshell: the presented workflow allows for a robust and objective diagnosis at least for tumorous and normal epithelial tissue.

## Conclusion

In the present study a combination of multimodal imaging and Raman-microspectroscopy is suggested as a fast and precise pathological screening tool. This combination of optical approaches bundles a fast overview technique (multimodal imaging) for identification of suspicious regions (red flags) that are diagnosed by a highly molecular sensitive but rather slow method (Raman-spectroscopy). By applying multivariate statistical methods the multimodal images could be converted into pseudo-HE stain images, which can be analyzed by a pathologist in the same manner as normal HE images. The comparison of pseudo-HE images derived from multimodal images with real HE images proofs that both HE staining and multimodal imaging (TPEF, SHG and CARS) feature similar information. This pseudo-HE stain image can be used by a pathologist to highlight suspicious areas and mark them with red-flags. These red-flag areas can be further analyzed with other techniques featuring a higher sensitivity than non-linear multimodal imaging. In this contribution, the slow but molecular selective technique Raman-spectroscopy was tested for a precise and robust diagnosis. Compared to a gold-standard diagnosis of an experienced pathologist, the Raman-based diagnostics featured 100 % mean sensitivity for the prediction of normal and tumor tissue. The differential diagnosis, the prediction of adenoma, carcinoma and normal epithelium, exhibits a mean sensitivity of around 80 %. Thus, further improvement is required if a differential diagnosis is desired. Nevertheless, the combination of multimodal imaging and Raman-microspectroscopy as a fast, reliable tool to screen large tissue areas and to diagnose normal epithelial tissue from malignant tissue (adenoma, carcinoma) could be demonstrated.

The presented combination of multimodal imaging and Raman spectroscopy can support a pathologist especially in a setting where the preparation of high quality fixated and embedded tissue sections is hardly possible, like for an intraoperative cryosection analysis. As both optical imaging methods are non-invasive, a subsequent staining with conventional HE stain remains feasible, allowing for a direct comparison with the current pathological gold-standard or other stains and methods. The non-invasive character of the methodology introduced within this article also allows for further *in-vivo* applications. The technical transformation of the combination of multimodal imaging and Raman-spectroscopy into an operation theater for *in-vivo* studies during an operation is possible and subject to current efforts of us. Such an *in-vivo* application would increase the possibilities of cancer diagnosis and treatment, since an online-monitoring of certain areas can be performed.

## Abbreviations

PCA, principal component analysis; SNIP, statistics-sensitive non-linear iterative peak-clipping; KKNN, weighted k-nearest neighbors; IO-CV, individual-out-cross-validation; NAD, Nicotinamide adenine dinucleotide; LDA, linear discriminant analysis model; OCT, optical coherence tomography; SHP, spectral-histo-pathology; CARS, coherent anti-Stokes Raman-scattering; TPEF, two-photon excited autofluorescence; SHG, second harmonic generation; HE, hematoxylin and eosin
